# Double-Breasted Anterior Rectus Sheath Turnover Flap

**DOI:** 10.7759/cureus.49302

**Published:** 2023-11-23

**Authors:** Wen Yang Chung, Koh Siang Chai, Daphne Stephen, John Ranjit

**Affiliations:** 1 Department of Plastic and Reconstructive Surgery, Sarawak General Hospital, Kuching, MYS; 2 Department of Plastic and Reconstructive Surgery, Subang Jaya Medical Centre, Subang Jaya, MYS

**Keywords:** surgical wound management, plastic surgergy, component separation technique, wound care and reconstructive surgerye, open abdomen surgery

## Abstract

The management of the open abdomen follows wound management with temporary abdominal closure prior to definitive closure while concurrently managing patient nutrient and fluid losses. This case report describes the successful use of double-breasted anterior rectus sheath turnover (DART) flap for early open abdomen closure to facilitate oncological management. The patient is a 47-year-old female with uterine smooth muscle neoplasm whose laparotomy wound was complicated with abdominal wound dehiscence and intra-abdominal infection. The abdomen could be closed with no fistula formation, iatrogenic bowel perforations, or overlying skin necrosis, and a follow-up showed no hernia occurrence. In conclusion, the DART flap provides a simple and autologous option for early tension-free midline closure of the open abdomen with acceptable intra- and postoperative complications.

## Introduction

The management of the open abdomen begins with wound management and temporary abdominal closure (TAC). Concurrently, the patient’s nutrition and fluid losses from the open abdomen are optimized. A definitive closure of the abdomen is then decided upon, be it immediate or delayed depending on the patient’s condition [1]. After eight days, tension-free midline closure is difficult due to soft tissue edema and lateralization of the abdominal musculature. By then, patients will be at an increased risk of open abdomen complications, such as fistulae, as high as 25% [2]. In this case report, we describe a technique of early closure of an open abdomen using double-breasted anterior rectus sheath turnover (DART) flap.

## Case presentation

A 47-year-old female with underlying diabetes, hypertension, and heart disease was diagnosed with uterine smooth muscle neoplasm. She underwent a total abdominal hysterectomy with bilateral salpingo-oophorectomy and bilateral pelvic lymph node sampling, which, unfortunately, was complicated by abdominal wound dehiscence. Due to intra-abdominal infection and collection, she underwent wound debridement and abdominal washout 10 days later. Tension-free primary fascial closure was not feasible as the bowels were edematous and rectus muscles displaced laterally. Therefore, the decision was made to treat it as an open abdomen.

The patient was then referred to the plastic surgical team for early abdominal wound closure to facilitate subsequent oncological treatment. As intraoperative tissue culture grew Escherichia coli with extended-spectrum beta-lactamases (ESBL), the patient was treated with local wound care and systemic antibiotics for another 10 days. Once the infection resolved, she was scheduled for abdominal wound closure with the DART flap.

Surgical technique

**Figure 1:**
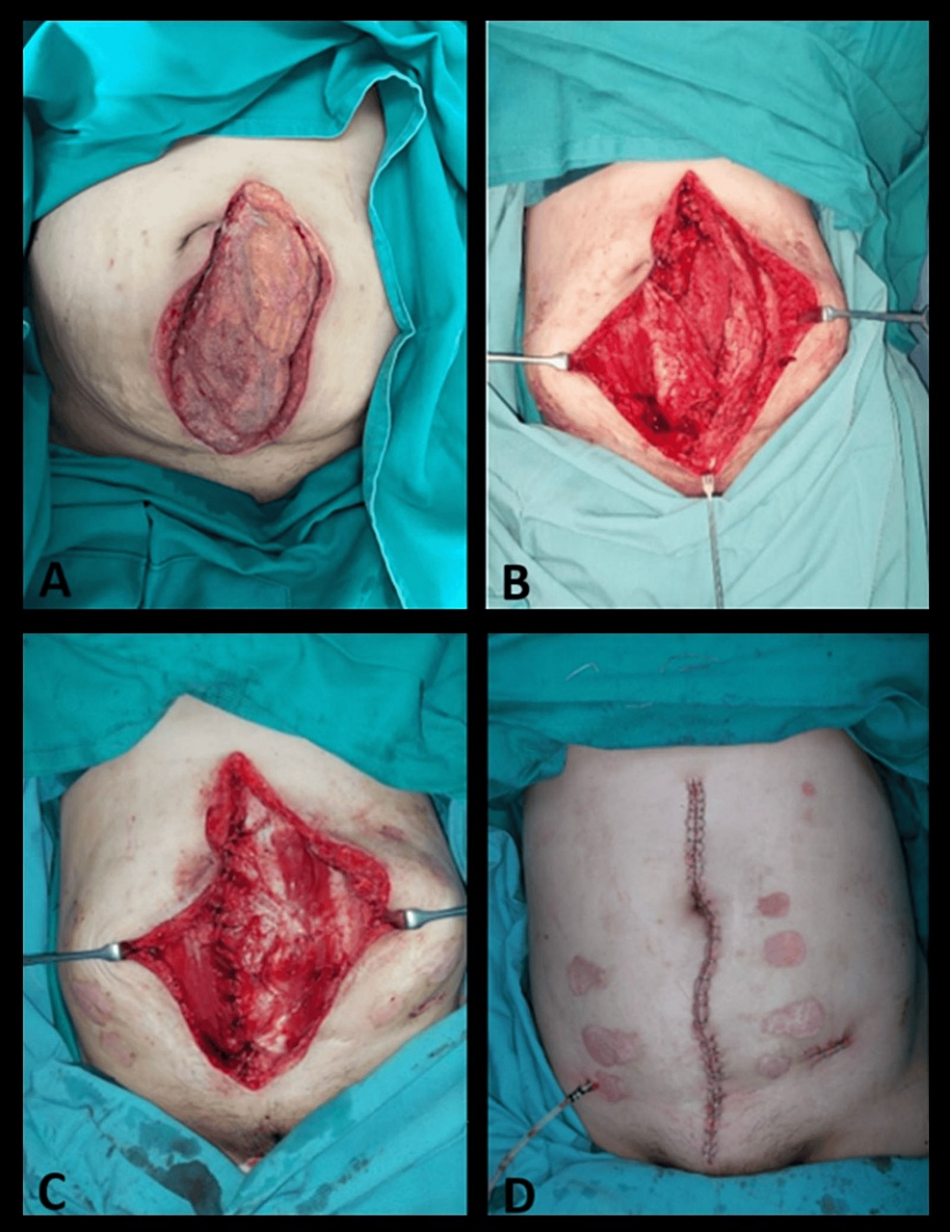
Intraoperative pictures of the DART flap (A) Abdominal contents covered with omentum (B) Right anterior rectus sheath raised and turned over (C) Left anterior rectus sheath raised, turned over, and sutured to the medial border of contralateral rectus abdominis muscle (D) Closure of abdominal wound DART, double-breasted anterior rectus sheath turnover

**Figure 2:**
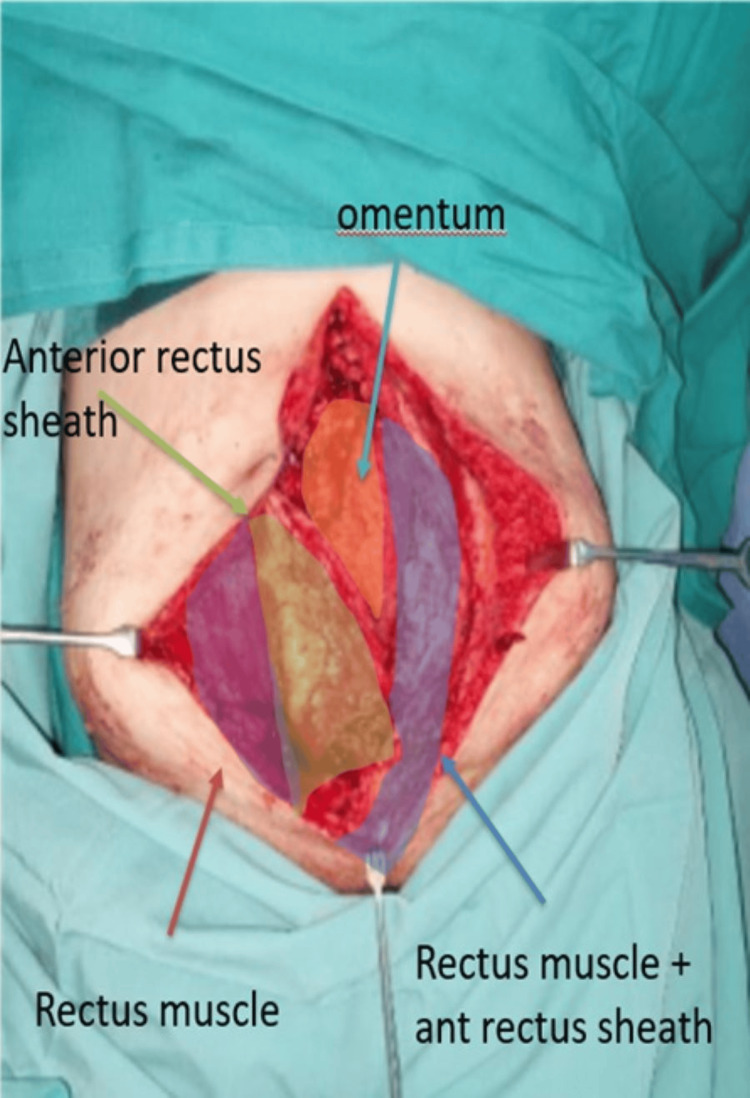
Legend of anatomical structure

**Figure 3:**
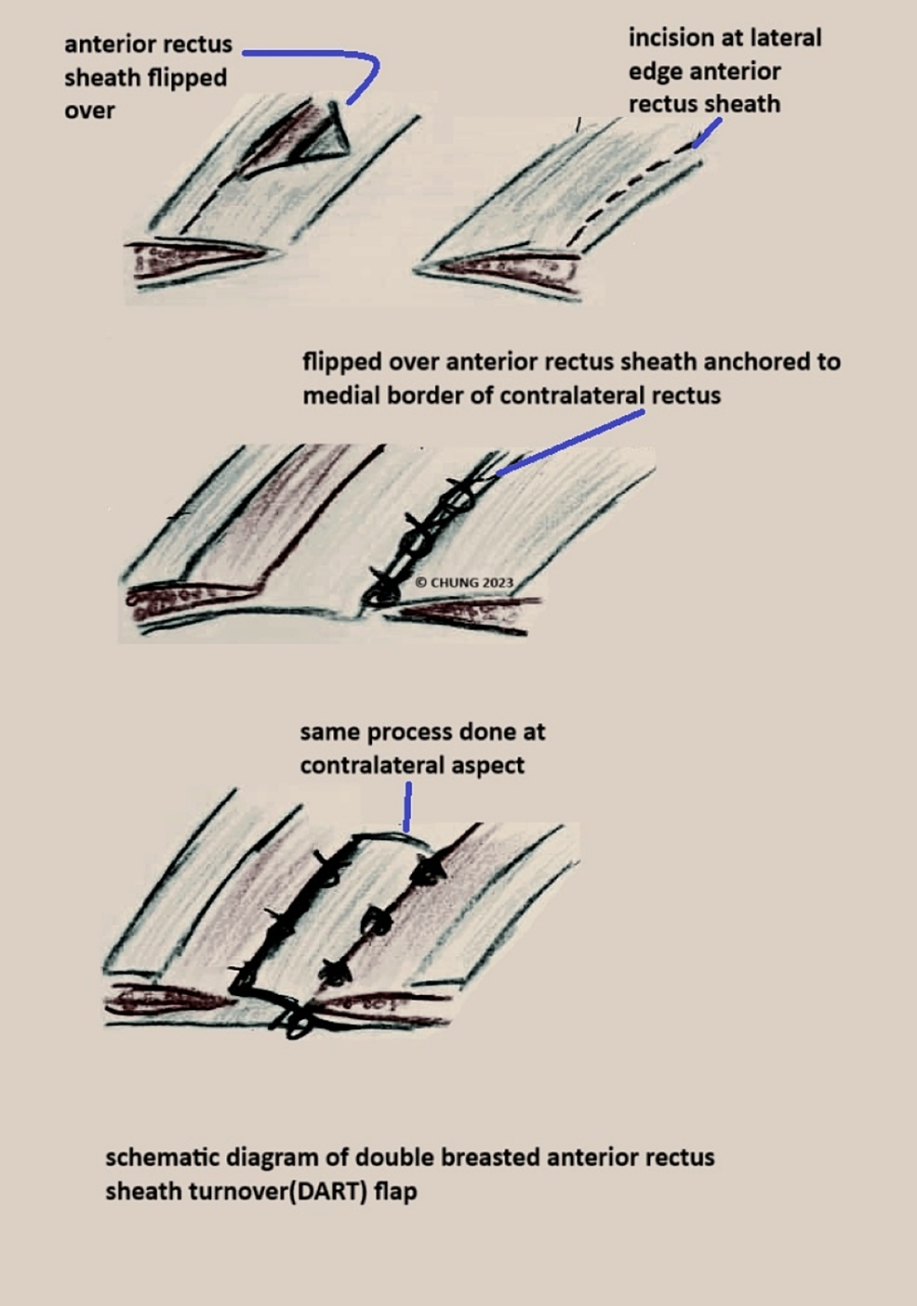
Schematic diagram of the DART flap DART, double-breasted anterior rectus sheath turnover

The abdominal wound defect measured 22 × 10 cm with indurated wound edges. The abdominal contents were covered by omentum with thin, overlying granulation tissue (Figure 1A). The rectus sheath and muscles were first identified. An incision was made 2 cm medially from the lateral border of the anterior rectus sheath along the length of the rectus muscle. The anterior rectus sheath was then separated from the underlying rectus muscle and turned over medially (Figure 1B). These steps were repeated on the contralateral rectus sheath. The free edge of the rectus sheaths was sutured to the medial border of the contralateral rectus muscles using absorbable sutures, forming a double-breasted sheath (Figure 1C). No synthetic mesh was placed to further strengthen the DART flap due to the recent infection. Drains were inserted, and overlying tissue was approximated and closed in layers (Figure 1D). Figures 2, 3 show the legend of anatomical structures and schematics diagram of the DART flap. 

Result

**Figure 4:**
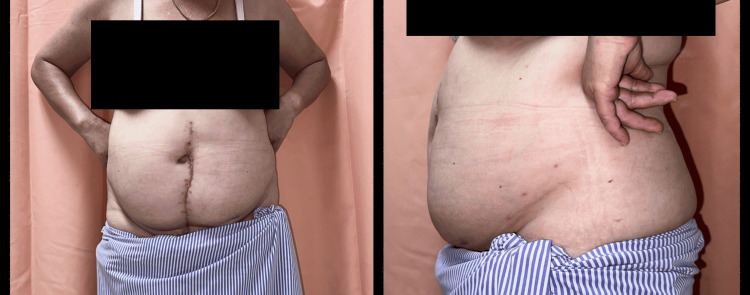
Two-year postoperative follow-up

Postoperative, the patient was advised to avoid abdominal straining and kept on the abdominal binder. Two weeks after surgery, the wound healed and drains and skin staplers were removed. The patient was allowed home with advice and on routine follow-up. Throughout two years of follow-up, no incisional hernia was detected (Figure 4).

## Discussion

Often, the primary surgeon would have to consider abdominal closure based on the need for second-look surgery, infection, and intra-abdominal hypertension. In the event whereby the primary closure is not feasible, TAC is used in forms such as Bogota bag or negative pressure wound therapy [1-3]. This delay often leads to soft tissue edema and lateralization of the abdominal musculature, preventing tension-free primary fascial closure. As such, techniques such as skin grafting and component separation have been used to close the abdomen [2].

Component separation by Ramirez in 1990 is a surgical technique used for separating the abdominal muscle components to allow for mobilization and, hence, tension-free midline abdominal closure. In anterior component separation, the extensive undermining of overlying skin often leads to skin necrosis. While in posterior component separation, the release of adhesions of abdominal content from the posterior rectus sheath is required, risking iatrogenic bowel perforations [4,5]. When paired with the known complications, this technique is not used for this patient in view of recent multiple abdominal surgeries and manipulation with recent infection leading to cocooning of the abdominal content and unreliable overlying skin vasculature.

Skin grafting with deliberate incisional hernia creation is also not a viable option for this patient. Such a method would first require adequate granulation tissue over the abdominal content. Despite the advancement of TAC methods such as negative pressure wound therapy, the patient would still require up to one month to allow for granulation [3]. This long duration of an open abdomen would hinder oncological management, hence delaying the management of malignancy.

In addition, this would also mean that the patient will require long-term intensive care admission, increasing the risk of nosocomial infections. The patient would also be subjected to an increased risk of open abdomen complications, such as fistulae, further complicating the wound [1]. The increased morbidity to the patient and long-term usage of advanced TAC methods would increase the financial burden and resources of both the patient and the healthcare system [3].

Another consideration to take into account when closing an open abdomen is the abdominal wall integrity. In this aspect, skin grafting with deliberate incisional hernia creation is least desirable. Patients often would have to undergo another surgery at a later date to manage the incisional hernia [2]. On the other hand, the anterior component separation method without mesh has been shown to have a high incidence of hernia occurrence [4]. Usage of synthetic mesh is also not advocated in cases of recent infection in lieu of high risk of implant infection.

The DART flap is an early open abdominal closure technique. It can be performed once the underlying infection has been resolved, reducing the need for prolonged hospitalization and the risk of nosocomial infections. The dissection is also minimal, reducing intraoperative complications such as iatrogenic bowel perforations. As the anterior rectus sheath is an autologous tissue, there is also a reduced risk of infection. The long-term follow-up from studies shows a low incidence of hernia occurrence when using the anterior rectus sheath turnover flap, proving its durability [6,7]. Unfortunately, the DART flap can only be used to close small to moderate defects. For larger defects, a combination of anterior rectus sheath turnover flap and component separation technique may be required.

This report focuses on the success of a single case, limiting its generalization to a broader population. The absence of comparative analysis and selection criteria further limits its practical usage. The follow-up of the patient of two years may not reflect on longer term complications and durability of this technique. Some improvements that can be made would be a larger sample size, comparative analysis, and longer follow-up period to further validate the advantages of using the DART flap in diverse clinical scenarios.

## Conclusions

Multiple factors must be taken into consideration when deciding on closing an open abdomen. The goals of any open abdominal closure should be to provide tension-free closure, reduction in morbidity to the patient, and restoration of abdominal wall integrity. The DART flap is a simple and autologous option for early tension-free midline closure of open abdomens with acceptable intra- and postoperative complications.
